# Circulating microRNAs found dysregulated in ex-exposed asbestos workers and pleural mesothelioma patients as potential new biomarkers

**DOI:** 10.18632/oncotarget.12408

**Published:** 2016-10-03

**Authors:** Ilaria Bononi, Manola Comar, Andrea Puozzo, Mariarita Stendardo, Piera Boschetto, Sara Orecchia, Roberta Libener, Roberto Guaschino, Silvia Pietrobon, Manuela Ferracin, Massimo Negrini, Fernanda Martini, Massimo Bovenzi, Mauro Tognon

**Affiliations:** ^1^ Department of Morphology, Surgery and Experimental Medicine, Section of Pathology, Oncology and Experimental Biology, School of Medicine, University of Ferrara, Ferrara, Italy; ^2^ Institute for Maternal and Child Health - IRCCS “Burlo-Garofolo”– Trieste, University of Trieste, Trieste, Italy; ^3^ Department of Medical Sciences, School of Medicine, University of Ferrara, Ferrara, Italy; ^4^ Mesothelioma BioBank, Pathology Unit and City Hospital, Alessandria, Italy; ^5^ Transfusion Medicine, City Hospital, Alessandria, Italy; ^6^ Department of Experimental, Diagnostic and Specialty Medicine – DIMES, University of Bologna, Bologna, Italy; ^7^ Laboratory for Technologies of Advances Therapies (LTTA), University of Ferrara, Ferrara, Italy; ^8^ Clinical Unit of Occupational Medicine, Department of Medical Sciences, School of Medicine, University of Trieste, Trieste, Italy

**Keywords:** microRNA, mesothelioma, biomarker, asbestos, worker

## Abstract

Malignant pleural mesothelioma (MPM), a fatal cancer, is an occupational disease mostly affecting workers ex-exposed to asbestos fibers. The asbestos, a cancerogenic mineral of different chemical composition, was widely employed in western Countries in industrial manufactures of different types. MPM may arise after a long latency period, up to five decades. MPM is resistant to conventional chemo- and radio-therapies. Altogether, these data indicate that the identification of new and specific markers are of a paramount importance for an early diagnosis and treatment of MPM. In recent years, microRNAs expression was found dysregulated in patients, both in cancer cells and sera, affected by tumors of different histotypes, including MPM. Cell and circulanting microRNAs, found to be dysregulated in this neoplasia, were proposed as new biomarkers. It has been reported that circulating microRNAs are stable in biological fluids and could be employed as potential MPM biomarkers. In this investigation, circulating microRNAs (miR) from serum samples of MPM patients and workers ex-exposed to asbestos fibers (WEA) and healthy subjects (HS) were comparatively analyzed by microarray and RT-qPCR technologies. Our results allowed (i) to select MiR-3665, an endogenous stable microRNA, as the internal control to quantify in our analyses circulating miRNAs; to detect (ii) miR-197-3p, miR-1281 and miR 32-3p up-regulated in MPM compared to HS; (iii) miR-197-3p and miR-32-3p up-regulated in MPM compared to WEA; (iv) miR-1281 up-regulated in both MPM and WEA compared to HS. In conclusion, three circulating up-regulated microRNAs, i.e. miR-197-3p, miR-1281 and miR-32-3p are proposed as potential new MPM biomarkers.

## INTRODUCTION

Malignant pleural mesothelioma (MPM) is a fatal cancer, with an increasing incidence worldwide. MPM, which is resistant to conventional chemo- and radio-therapies, is often diagnosed in a late stage with a median survival of 12 months. Exposure to asbestos fibers is the main risk factor for the MPM onset [1], which may occur in workers even decades after exposure to this tumorigenic mineral. Recent data indicate that asbestos is becoming an environmental pollutant, with the consequence that MPM patients not always are linked to the occupational disease [2]. Asbestos, after been recognized as a carcinogenic agent [3], was banned in different European Countries at various time-points between 1970-2005 [4, 5]. It was estimated that about 250,000 people will die of this tumour in Europe in the coming decades [6, 7].

Considering the long latency period of MPM onset, subjects potentially at risk may benefit of an early diagnosis based on specific biomarkers [8]. Many studies attempted to identify specific biomarkers that may predict the evolution of this malignancy without firm conclusions [9–12]. Indeed, the identification of new and specific markers is of a paramount importance for an early detection, diagnosis, and treatment of MPM, together with the evolution of this malignancy [13]. In recent years, together with protein markers, microRNAs (miRNAs) from MPM cells or sera, have been proposed as new biomarkers [14–16]. MiRNAs, a family of small non-coding RNAs, approximately 21-25 nt long, negatively regulate the gene expression by inhibiting translation of target messenger RNAs (mRNAs) through pairing with mRNA recognition elements (MREs), usually in 3′-UTRs [17]. Circulating miRNAs are packaged in microparticles, such as exosomes, microvesicles and apoptotic bodies or by their association with RNA binding protein including Argonaute 2 (Ago2) or lipoprotein complexes like the high-density lipoprotein (HDL), with the final effect of enhancing their stability in biological fluids [18]. It was hypothesized that these miRNAs detectable in serum samples, may allow to study the inter-cellular information flow [19–21].

Dysregulated miRNAs have been detected in several cancers of different histotypes [22]. Many investigations indicate that miRNAs may be used as diagnostic biomarkers for cancers, including MPM [23, 24] and potential new targets for innovative therapeutic approaches [25].

In a previous study we identified a group of 22 miRNAs significantly dysregulated in MPM cells compared to normal mesothelial cells. Some of these miRNAs belong to miR-17-92 cluster, which is induced by c-Myc oncogene [14].

Herein, we report on the comparative analysis of miRNA expression in serum samples from MPM affected patients and workers ex-exposed to asbestos fibers (WEA) and healthy subjects (HS). The aim of this investigation was to identify extracellular miRNAs as (i) putative biomarkers for MPM; (ii) predictive markers of MPM in WEA; (iii) potential targets for innovative therapies.

## RESULTS

### Microarray analysis

In our investigation, circulating miRNAs from serum samples of MPM patients (n=10) and workers (n=10), both groups belonging to ex-exposed asbestos fibers, and healthy subjects (n=10), were analysed by microarray (Agilent Technologies, Human miRNA microarray G4470A) and RT-qPCR technologies.

**Figure 1:**
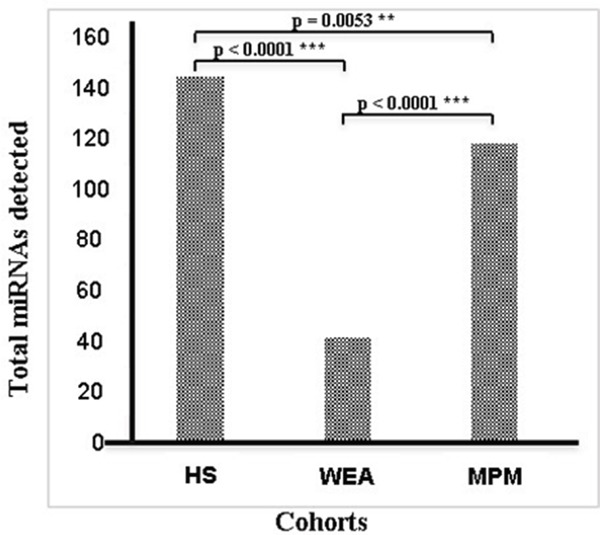
Total number of miRNAs detected in the three cohorts MPM, WEA and HS analyzed by microarray technology The difference between: HS and MPM, i.e 145 vs 119, p=0.053; HS vs WEA, i.e. 145 vs 42, p<0.0001; MPM vs WEA, i.e. 119 vs 42, p<0.0001, are statistically significant.

Among the 1,201 miRNAs assessed with microarray technology, 197 miRNAs were identified to be detected in at least 1 out of 30 samples analyzed. It is worth noting that the total number of circulating microRNAs in the three cohorts was different. Indeed, differences in miRNA expression profiles of MPM, WEA and HS were identified, with more miRNAs detected in serum of healthy subjects compared to that of MPM and WEA. Specifically, 145 miRNAs out of 1,201 were detected in healthy subjects, whereas they were 119 in MPM and 42 in WEA. The difference between: HS and MPM, i.e 145 vs 119, p=0.053; HS vs WEA, i.e. 145 vs 42, p<0.0001; MPM vs WEA, i.e. 119 vs 42, p<0.0001, are statistically significant (Figure 1).

**Figure 2:**
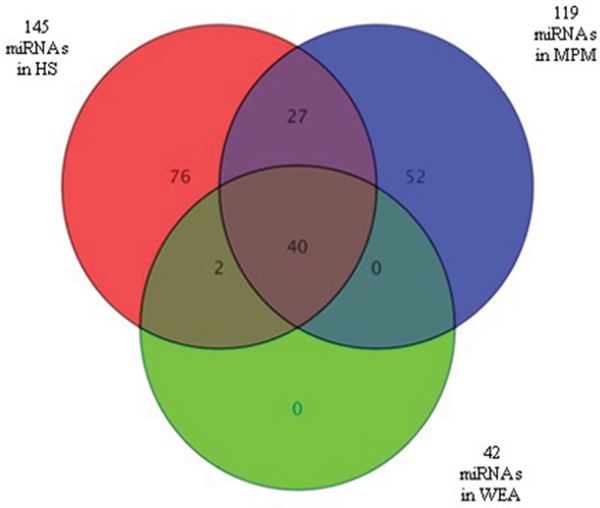
Venn diagram showing miRNAs detected in sera from the three MPM, WEA, HS cohorts analyzed MiRNAs (n=119) expressed in MPM are in blue, n=42 are those detected in WEA cohort, in gree, whereas n=145 are the miRNAs detected in HS, in red. MiRNAs n=52 are expressed in MPM samples only. N=76 are expressed in the HS only. None of the MiRNAs (n=42) detected in the WEA cohort was unique of this cohort. Indeed, n=40 were in common with MPM and HS, n=2 were in common with HS only. N=27 miRNAs were found in common between MPM and HS.

Among the 119 miRNAs expressed in MPM, 67 miRNAs were also detected in the control sample of HS. The remaining 52 miRNAs were expressed only in the MPM samples. In the WEA cohort 42 miRNAs were expressed, but none was expressed exclusively in this cohort. A total of 145 miRNAs were expressed in HS, i.e. 76 miRNAs were detected in this cohort only, whereas 27 were in common with MPM, 2 were in common with WEA and 40 were expressed in all the three groups of patients/subjects studied (Figure 2).

**Figure 3:**
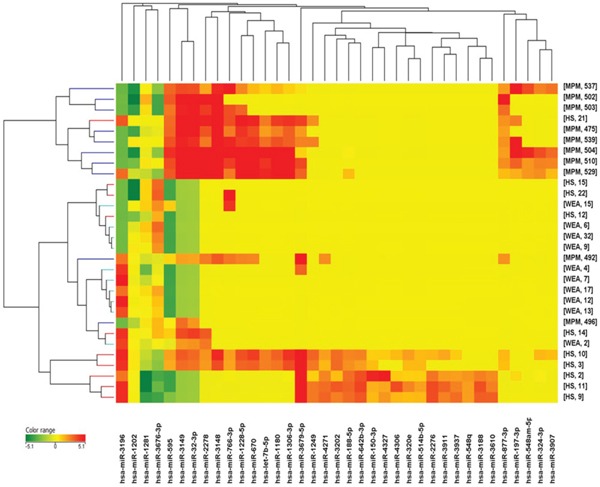
Cluster analysis of microRNA expression profiles in sera from MPM, WEA and HS cohorts

Dysregulated miRNA expression was detected in serum samples from MPM patients, compared to WEA and HS. Comparing with ANOVA test the miRNAs expression in the three groups (MPM, WEA and HS) 37 miRNAs were found to be significantly dysregulated (Figure 3 and Table 1).

**Table 1:** MiRNAs detected dysregulated in three groups by microarray analysis

n	Systematic name	Normalized data			MPM vs HS		MPM vs WEA		WEA vs HS		False discovery rate
		MPM	WEA	HS	Regulation	FC	Regulation	FC	Regulation	FC	
1	let-7b-5p	−1,1021	−3,2156	−2,4594	up	2,5620	up	4,3271	down	−1,6890	5,25
2	miR-1180	−0,6633	−3,2156	−2,2398	up	2,9824	up	5,8656	down	−1,9667	5,08
3	miR-1202	1,2882	4,1715	2,5602	down	−2,4151	down	−7,3785	up	3,0552	8,17
4	miR-1228-5p	0,3912	−3,2156	−2,0449	up	5,4117	up	12,1823	down	−2,2511	0,70
5	miR-1249	−3,0104	−3,2156	−1,7707	down	−2,3615	up	1,1528	down	−2,7223	1,90
6	miR-1281	3,4131	3,2472	1,5335	up	3,6796	up	1,1218	up	3,2800	9,80
7	miR-1306-3p	−0,6182	−3,2156	−2,0092	up	2,6226	up	6,0519	down	−2,3076	7,16
8	miR-150-3p	−3,2695	−3,2156	−2,2309	down	−2,0542	down	−1,0381	down	−1,9788	5,16
9	miR-188-5p	−3,0793	−3,2156	−1,8494	down	−2,3454	up	1,0991	down	−2,5778	1,90
10	miR-197-3p	−0,7610	−3,2156	−2,9142	up	4,4480	up	5,4815	down	−1,2323	4,75
11	miR-2276	−3,2695	−3,2156	−2,0743	down	−2,2898	down	−1,0381	down	−2,2058	4,75
12	miR-2278	0,7015	−2,9345	−1,9823	up	6,4258	up	12,4323	down	−1,9347	0,22
13	miR-3148	1,8715	−3,2156	−1,8038	up	12,7759	up	33,9906	down	−2,6605	0,01
14	miR-3149	3,3830	−2,8135	−0,9095	up	19,5963	up	73,3389	down	−3,7425	0,01
15	miR-3188	−3,2695	−3,2156	−2,3036	down	−1,9532	down	−1,0381	down	−1,8815	8,37
16	miR-3196	−2,1135	0,9510	1,5813	down	−12,9485	down	−8,3654	down	−1,5479	8,17
17	miR-32-3p	3,1347	−2,8072	−1,0024	up	17,5959	up	61,4774	down	−3,4939	0,01
18	miR-3202	−3,2695	−3,2156	−1,9073	down	−2,5708	down	−1,0381	down	−2,4765	1,43
19	miR-320e	−3,2695	−3,2156	−2,4206	down	−1,8011	down	−1,0381	down	−1,7351	1,52
20	miR-324-3p	−2,1411	−3,2156	−3,0620	up	1,8933	up	2,1059	down	−1,1123	7,31
21	miR-3610	−3,2695	−3,2156	−2,4331	down	−1,7856	down	−1,0381	down	−1,7201	8,37
22	miR-3676-3p	−0,9013	1,8540	−0,0857	down	−1,7600	down	−6,7518	up	3,8363	4,75
23	miR-3679-5p	−1,3530	−2,8888	0,2802	down	−3,1020	up	2,8996	down	−8,9947	4,75
24	miR-3907	−2,2813	−3,2156	−3,1256	up	1,7953	up	1,9109	down	−1,0644	7,44
25	miR-3911	−3,2695	−3,2156	−2,2902	down	−1,9715	down	−1,0381	down	−1,8991	4,69
26	miR-3937	−3,2695	−3,2156	−2,3920	down	−1,8371	down	−1,0381	down	−1,7697	4,75
27	miR-4271	−3,0353	−3,2156	−2,0329	down	−2,0033	up	1,1331	down	−2,2699	4,75
28	miR-4306	−3,2695	−3,2156	−2,4794	down	−1,7291	down	−1,0381	down	−1,6657	7,51
29	miR-4327	−3,2695	−3,2156	−2,2034	down	−2,0936	down	−1,0381	down	−2,0169	9,69
30	miR-514b-5p	−3,2695	−3,2156	−2,5183	down	−1,6831	down	−1,0381	down	−1,6214	1,10
31	miR-548am-5p	−2,0017	−3,2156	−3,2610	up	2,3938	up	2,3196	up	1,0320	5,08
32	miR-548q	−3,2695	−3,2156	−2,6473	down	−1,5391	down	−1,0381	down	−1,4827	8,17
33	miR-595	3,3451	−2,4018	−0,8586	up	18,4264	up	53,7046	down	−2,9145	0,01
34	miR-642b-3p	−3,2695	−3,2156	−1,6936	down	−2,9811	down	−1,0381	down	−2,8717	1,36
35	miR-670	−0,1444	−3,2156	−2,0461	up	3,7365	up	8,4044	down	−2,2492	3,14
36	miR-766-3p	0,1875	−2,7920	−1,9239	up	4,3209	up	7,8871	down	−1,8253	3,35
37	miR-877-3p	−0,9961	−3,2156	−2,7473	up	3,3664	up	4,6572	down	−1,3835	0,03

### Validation by RT-qPCR of stable circulating miRNAs

The normalization of data is a prerequisite to verify whether false negative/positive results may occur. In addition, this normalization is necessary to reduce some of the potential technical variations, such as the kind of sample, the method of RNA extraction, miRNA quantification methods. To this purpose in our experiments, endogenous stable miRNAs were investigated. Herein, microarray results indicated three endogenous unvariable expressed miRNAs, i.e. miR-1234-3p, miR-3656 and miR-3665. Specifically, these three candidate control miRNAs were selected because showing the less expression variability in microarray analysis across all analyzed sera.

Then, RT-qPCR technique was employed to validate the data on the three stable miRNAs obtained with the microarray approach. These RT-qPCR analyses were carried out in the 30 sera (n=10 MPM, n=10 WEA and n=10 HS), analyzed before by microarray technology, together with additional 19 serum samples from MPM, n=10; WEA, n=5; HS, n=4, for a total of 49 sera.

In order to verify whether the three candidate stable miRNAs could be used as controls, the average coefficient of variation (CV), across the three different cohorts, was evaluated. ANOVA test was employed to study the differences in mean ΔCT of the three candidate stable miRNAs in the three MPM, WEA and HS groups. Moreover, the NormFinder software was used to verify the S parameter, which is the relative value indicating the stable miRNA expression. Three values, i.e. CV, ANOVA and S parameter, were employed to select the more stable microRNA among the 49 samples analyzed. CV values resulted, 0.035%, 0.023% and 0.023% for miR-1234-3p, miR-3656 and miR-3665, respectively. For miR-1234-3p ANOVA test showed p = 0.81, for miR-3656 resulted p = 0.57 and p = 0.81 for miR-3665. S parameter resulted, 0.024, 0.027, 0.021 for miR-1234-3p, miR-3656 and miR-3665, respectively. Statistical analyses of microarray and RT-qPCR data showed no different expression for these three miRNA among the three cohorts. Although RT-qPCR confirmed the stable expression for the three candidate stable miRNAs, miR-3665 demonstrated the minimum CV, the maximum ANOVA value and the minimum S value, as expected for a significant data. Therefore, miR-3665 demonstrated the higher stable expression in our samples. This result led us to select miR-3665 as stable endogenous control for circulating miRNAs quantification in our cohorts. Moreover, this miRNA was selected as housekeeping to perform the relative quantification of miRNAs differentially expressed by the use of the value of fold changes (FC) calculated using the equation:

2^−Δ(ΔCt)^, where ΔΔCt=(Ct_miR_-Ct_HKmiR-3665_)_MMP_-(Ct_miR_-Ct_HKmiR-3665_)_HS_.

It is worth noting that until now, no HK standard for the circulating miRNA studies was identified in different studies carried out in this field. In our investigation an internal miRNA control, miR-3665, was selected. Indeed, this endogenous housekeeping gene showed consistently the same expression level in normal and patient samples.

### Validation of dysregulated circulating miRNAs by RT-qPCR

The validation of microarray data was performed by RT-qPCR on three miRNAs that were differentially expressed in the three different cohorts MPM, WEA and HS. The results are shown in Tables 2, 3 and 4.

**Table 2:** Microarray data of differentially expressed miRNAs miR-197-3p miR-1281 and miR-32-3p

Systematic name	Comparison	Regulation	FC	ANOVA p
miR-197-3p	MPM vs HS	UP	4.4	0.047
	MPM vs WEA	UP	5.5	0.047
miR-1281	MPM vs HS	UP	3.7	0.097
	WEA vs HS	UP	3.3	0.097
miR-32-3p	MPM vs HS	UP	17.6	<0.001
	MPM vs WEA	UP	61.5	<0.001

**Table 3:** RT-qPCR data of differentially expressed miRNAs miR-197-3p and miR-1281. The reported ANOVA value shows the statistical significance of the three cohorts, and the t-test value indicating the statistical significance in each comparison between two groups to underline major differences

Systematic name	Comparison	Regulation	FC	ANOVA p	t-tesp p
miR-197-3p	MPM vs HS	UP	1.8	0.0240*	0.0392*
	MPM vs WEA	UP	2.5		0.0194*
miR-1281	MPM vs HS	UP	2.5	0.0061**	0.0121*
	WEA vs HS	UP	3.5		0.0.124*

**Table 4:** RT-qPCR data of differentially expressed miRNAs miR-32-3p

Systematic name	Comparison	Regulation	Pos/tot (% pos)	t-test p
miR-32-3p	MPM vs HS	UP	6/20 (30%) MPM 0/16 (0%) HS	0.024*
	MPM vs WEA	UP	6/20 (30%) MPM 0/15 (0%) WEA	0.027*

The three miRNAs, miR-197-3p, miR-1281 and miR-32-3p, were found up-regulated with both techniques, microarray and RT-qPCR, in MPM compared to HS. MiR-197-3p and miR-1281 were detected in all sera analyzed (n= 49). This data allowed us to calculate the difference in average of 2^−ΔCt^ by ANOVA, t-test and the FC.

**Figure 4:**
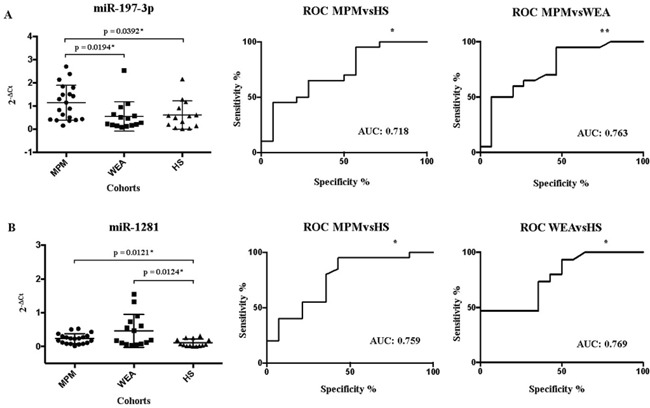
Schematic representation of the Receiver Operating Characteristic (ROC) In Panel A the MiR-197-3p expression in MPM, WEA and HS cohorts in the X axis, detected by RT-qPCR, is represented as 2^−ΔCt^ value in the Y axis. The ROC AUC for miR-197-3p ratio MPM vs HS, p = 0.0328*; 95% confidence interval 0.5398-0.8959, and MPM vs WEA, p = 0.0085**; 95% confidence interval 0.5981 to 0.9285. Panel B: MiR-1281 expression in sera from MPM patients, WEA and HS, by RT-qPCR. The ROC AUC for miR-1281 ratio MPM vs HS, p = 0.0112* 95% confidence interval 0.5885-0.9293 and confrontation WEA vs HS, p = 0.0137*; 95% confidence interval 0.5963-0.9418. The statistically significant p values are indicate with * and **.

MiR-197-3p 2^−ΔCt^ value found in WEA and HS groups was similar, 0.548 and 0.616, respectively, while these two 2^−ΔCt^ values are statistically different from 2^−ΔCt^ value of 1.139 detected in the MPM group (Figure 4 – Panel A).

MiR-197-3p showed a different expression in the three cohorts, which is statistically significant by ANOVA, with a p= 0.0240. Then, the comparisons between MPM vs HS and MPM vs WEA gave p=0.0392 and p= 0.0194 by t-test, and FC=1.8 and FC= 2.5, respectively. Both comparative analyses gave p and FC values statistically significant. (Table 3).

MiR-1281 was detected with 2^−ΔCt^ value in MPM and WEA of 0.232 and 0.464, respectively. These two values differ statistically from 2^−ΔCt^=0.108 found in the HS group (Figure 4 – Panel B). The statistical analysis showed that the expression of this miRNA was different in the three cohorts by ANOVA with a statistically significant p=0.0061. Comparing MPM vs HS, the t-test gave a p=0.0121, and FC=2.5, both statistically significant. WEA vs HS comparison showed by t-test a p= 0.0.124 and the FC=3.5, once again these two values are statistically significant (Table 3).

MiR 32-3p was detected by RT-qPCR in only 6/49 analyzed sera. The low prevalence of 12% of this miRNA did not allow us to calculate the fold difference and the p-value. Specifically, miR32-3p was detected in 6/20 (30%) MPM sera, but it was absent in WEA and HS sera. (Table 4).

## DISCUSSION

### Circulating miRNA expression in MPM sera has been poorly investigated

In our study, high differences in miRNA expression profiles in MPM, WEA and HS were identified. (Figure 1 and 2). Significantly, more miRNAs were expressed in the control HS samples than in MPM and WEA sera, while the differences in the number of miRNAs detected in the three groups are statistically significant. This result is in agreement with previous studies on microRNAs and human cancers in general, including MPM. Authors detected a general down-regulation of miRNAs in tumors compared to normal tissues [26]. Moreover, similar results were reported in a MPM study. These data showed higher miRNA expression profiles in tumors vs. the normal tissues [27].

In our investigation miRNA expression profiles were analyzed in MPM, WEA and HS sera. It turned out that miRNA expression profiles in MPM account for 119 miRNAs. Interestingly 52 out of 119 miRNA were found expressed in MPM only, while they were absent in WEA and HS cohorts. In addition, our data showed that in WEA 42 miRNAs only were detected. However, 40 out of 42 miRNAs were also detected in the MPM and HS profiles, whereas 2 out of 42 miRNAs were present in the HS profiles. Considering that the exposure to asbestos fibers may induce dysregulation of the immune system [28–30], one may speculate that the asbestos fibers in WEA subjects induces miRNA gene silencing. This may explain a reduced number of miRNAs found in this cohort.

In our study, three miRNAs namely miR-197-3p, miR-1281 and miR-32-3p were found to be dysregulated. This result is strengthened by the identification and selection of the stable miR-3665 as the endogenous control for quantifying circulating miRNAs in MPM, WEA and HS cohorts. MiR-197-3p was resulted up-regulated in MPM vs HS with the fold-change of 4.4, whereas it was detected up regulated in MPM vs WEA with the fold-change of 5.5, by microarray analysis. This result is in agreement with the RT-qPCR data, which show lower ΔCT value in MPM serum groups in comparison with both WEA and HS cohorts. Similarly, miR-197-3p was detected significantly up-regulated in sera or tissues of a variety of human cancer: lung, breast, pancreatic, ovarian, follicular thyroid cancer, SCLC, and NSCLC [31–37].

The strongly up-regulation of miR-197 found in MPM sera in comparison with HS and WEA cohorts indicates that miR-197 is normally present in sera of HS and WEA. Thus, it is possible that asbestos fibers penetrated in the lung/mesothelium do not affect the release of this miRNA. Subsequently, after the MPM onset the release of this miRNA may increase, allowing the detection of miR-197 up-regulated. If confirmed by other studies this result is particularly significant because miR-197 could be a new specific biomarker of this neoplasia.

The microRNA.org database indicates the FOXO3 gene as a predictive target of miR-197. Interestingly, FOXO is a key gene promoting the apoptosis process. Based on this data, one may speculate that mir-197 upregulated could lead to the FOXO gene down-regulation, which in turn could block the apoptosis of the cancer cells.

MiR-1281 was found up-regulated in MPM vs HS with fold-change of 3.7. Comparative analysis between WEA with HS sera showed a fold-change 3.3, by microarray technique. These results were confirmed by RT-qPCR, with a lower ΔCT values in MPM and WEA groups in comparison with HS cohort. MiR 32-3p was investigated in human cancers by three different teams. Our data on MPM differ from previous results obtained in bladder cancer, where miR 32-3p was found down-regulated [38], in adrenocortical tumors it was not differentially expressed [39] and in non-^131^I-avid lung metastases of papillary thyroid carcinoma was up-regulated, compared to in ^131^I-avid lung metastases [40].

MiR-32-3p resulted up-regulated in MPM vs HS group, with a the highest fold-change of 18 in microarray. Moreover, comparative analyses MPM vs WEA showed this miR up-regulated with a fold-change of 61. The two results were confirmed by RT-qPCR: in particular with this technique we evidenced the expression of this miRNA only in 6 individuals of the MPM group and in none of WEA and HS groups. In another study the expression of miR-32-3p was found up-regulated in HCC and derived cell lines. [41]. It has been published that miR-32-3p down-regulates phosphatase and tensin homolog (PTEN) through binding to 3′-UTR of PTEN mRNA, whereas PTEN was identified as a tumor suppressor found mutated in a large number of cancers. These data indicate that the expression level of miR-32-3p could affect the proliferation, migration, and cancer cell invasion. Altogether these results suggest that *miR-32-3p* can be proposed as a potential target for cancer treatment [41]. The expression of miR-32-3p has been shown to be upregulated in malignancies of different histotypes, including kidney [42] and colorectal carcinomas (CRC) [43]. Jalava et al demonstrated that has-miR-32-3p targeted B-cell translocation gene 2 (BTG2), a transcriptional cofactor with anti-proliferative properties [44]. These data suggest that miR-32-3p has a fundamental role as an oncogene. Currently, there are accumulating evidences that the aberrant expression of this miRNA is linked to the development of different malignancies [43].

It has been proposed that circulating microRNAs can be used as new biomarkers. It has been suggested that these molecules could act at a distance, moving through the circulatory stream. Specific miRNAs can be incorporated into biological structures, which may protect them from degradation [18]. It was demonstrated that exosomes contain microRNA, which can be delivered to another cell, and can be functional in this new location [45–47]. One may speculate that the up or down regulation of circulating miRNAs is not linked to a variation of their synthesis within the cells, but they are probably related to the release activities into the blood stream.

In HS and WEA sera both miR-197 and miR-32-3 are expressed at low lever or even absent. In MPM sera an enhancement of expression of both these miRNAs is seen. Considering that miR-197 down-regulates FOXO3 gene, while miR-32-3p downregulates the tumor suppressor gene PTEN and the anti-proliferative factor BTG2, these events may participate to the MPM onset.

In conclusion, in our experiments a stable miRNA was detected and used as endogenous internal control (miR-3665), whereas three circulating miRNAs, miR-197-3p, miR-1281 and miR-32-3p, were found up-regulated in MPM compared to HS sera. These three miRNAs could function as oncogenes by promoting cell cycle progression and cell mobility in MPM as shown before in other human cancers. At the same time, these miRNAs probably regulate negatively the expression of tumor suppressor mRNAs by binding to their 3′UTR. This mechanism could generate a positive feedback loop that contributes to cancerogenesis.

Up-regulated miR-197-3p, miR-1281 and miR-32-3p are proposed as new biomarkers for MPM and potential targets for innovative cancer treatments. In addition, we may speculate that these three dysregulated miRNAs can be employed in the group of WEA subjects as markers to assess/predict over time the risk of the MPM onset.

## MATERIALS AND METHODS

### Serum samples

Serum samples were collected from patients affected by malignant pleural mesothelioma (MPM) and workers both ex-exposed to asbestos fibers (WEA) and healthy subjects (HS) at the Clinical Laboratory Analysis of the University Hospital of Ferrara, Occupational Medicine Unit of the University of Ferrara, and City Hospital of Alessandria. Sera were from discarded laboratory analysis samples, after routine analyses, before the incineration. Anonymously collected sera were coded with indications of age, gender and pathology only. Informed written consent was obtained from all patients / subjects. The project was approved by the Ethics Committee, Ferrara. Italy. Sera, in small aliquots, were stored at −80°C until the time of the analysis. MicroRNAs analysis by microarray technology was performed on 30 serum samples (n=10 MPM, n=10 WEA and n=10 HS, with median age of 64 years), whereas real time quantitative PCR (RT-qPCR) was extended to additional 19 sera, 49 samples total (n=20 MPM, n=15 WEA and n=14 HS, with median age of 65 years).

### RNA extraction

Total RNA, including microRNAs, was extracted from 200 μl of serum using miRNeasy Mini Kit (QIAGEN cod. 217004) according to manufacturer protocol. The final amount of miRNA, extracted from serum, may be influenced either miRNA extraction efficiency or RT-qPCR robustness (i.e. presence of inhibitors). These factors can be verified by synthetic non-human miRNAs added to the serum sample, employed as controls, before the RNA isolation. In order to adjust these parameters, synthetic cel-miR-39 miRNA (2.5 μl of 5 nM) was added to the serum sample (200 ul), soon after adding the Qiazol Reagent (1 ml). RNA was eluted in 30 μl RNAse-free water.

### MicroRNA microarray

MicroRNA expression profile was analyzed in 30 samples, n=10 MPM, n=10 WEA and n=10 HS, using the Agilent miRNA microarray (G4870A) technology. This array is capable to assess the expression of 1,201 human miRNAs. A constant volume of 8 μl RNA was employed for the hybridization procedure. Experiments were performed as previously described [14, 48]. Microarray raw data were analyzed using GeneSpring GX 13 software (Agilent Technologies). A quantile normalization was applied before statistical analyses. A signal was detectable (i.e. above background) in at least one sample for 197 miRNA probes.

### Microarray data analysis

MicroRNAs, differentially expressed in the three groups (MPM, WEA and HS) were identified having a 1.5 fold differential expression and a false discovery rate < 10% in ANOVA statistical analysis (GeneSpring software).

### Reverse transcription

Reverse transcription reactions (10 μL) were performed using miRCURY LNA Universal RT microRNA PCR (EXIQON: cod EX203301). Two μL of total RNA was reverse transcribed after adding 0.5 μL of Synthetic RNA spike-in UniSp6, as an exogenous control. Reactions were carried out on the PTC-100 thermal cycler (MJ Research) using the following conditions: 42°C for 60 min, 95°C for 5 min, and then hold at 4°C. RT products were stored at −20°C until the time of the real-time PCR analysis. Cel-miR-39 assay was performed to monitor RT reaction efficacy.

### RT-qPCR analysis of endogenous miRNAs

Reverse Transcription Quantitative PCR (RT-qPCR) analysis was performed to validate three differentially expressed miRNAs from microarray analysis. These miRNAs were miR-1281, miR-197-3p, and miR 32-3p. In addition, three reference miRNAs, miR-1234-3p, miR-3656 and miR-3665, which showed stable expression, were valided. This approach was employed with the aim to select stable endogenous controls to quantify the circulating miRNAs. RT-qPCR reactions were performed using a ExiLENT SYBR® Green master mix (EXIQON) according to the manufacturer's instructions. Briefly, 1:40 diluted cDNA template was added to PCR primer mix and PCR Master mix, in 10 μL. Samples were run in triplicate (CFX96 Touch Real-Time PCR Detection System, Applied Biosystems) with denaturation at 95°C 10 min, followed by 40 cycles at 95°C 10 s, 60°C 1 min. A final melting curve analysis, from 60°C to 95°C, was carried out to verify the presence of a single amplified product.

### Determination of reference genes

For each candidate reference miRNA, the expression variability was assessed by calculating the overall coefficient of variability (CV). Each candidate endogenous control miRNA was tested in triplicate in RT-qPCR and the results expressed as mean Ct. To confirm the invariability we used also two statistical methods ANOVA test and NormFinder (http://moma.dk/normfinder-software); this software evaluted the stability and rank of each candidate house-keeping miRNA. The algorithm of NormFinder uses a mathematical model and a solid statistical framework to calculate the gene expression stability. NormFinder estimates the overall expression validation of the candidate normalizer genes, as well as the intra-group and the inter-group variations.

### Validation of circulating miRNAs expression

The level of three miRNAs, miR-1281, miR-197-3p and miR 32-3p, which resulted differentially expressed according to microarray analysis, was measured by RT-qPCR. MiRNA expression level was measured using the ΔCt method, where the Ct threshold cycle is the fractional cycle number when the fluorescence of each sample passes a fixed threshold. Relative quantification of each miRNAs was performed using as a references gene (HKmiR) a chosen stable endogenous control miRNA, which shows stably expression in all sera. The results were expressed as ΔCT (Ct_miR_-Ct_HKmiR_) for each subject and as mean ± S.D. of ΔCT for each group. High miRNA ΔCT value corresponded to low miRNA expression. The fold changes in relative miRNA expression were calculated using the equation 2^−Δ(ΔCt)^, where

ΔΔCt = (Ct_miR_-Ct_HKmiR_)_MMP_ - (Ct_miR_-Ct_HKmiR_)_HS_.

MiRNA species that were not detected in any of samples or with a CT value >39 were excluded from the comparison. MiRNA species with at least a 1.5-fold expression change among groups were considered differentially expressed.

Receiver Operating Characteristic (ROC) analysis was used to quantify the accuracy of a RT-qPCR result in discriminating comparative data of MPM vs HS, MPM vs WEA and WEA vs HS. The resulting ROC curve was generated by plotting true positive rate against false positive rate, at different threshold settings. The area under the curve (AUC) represents the probability that a random MPM/WEA is ranked as higher than a randomly chosen WEA/HS, the control. The highest AUC value = 1 means that the test perfectly discriminates MPM/WEA from the control, either WEA or HS.

### Statistical analysis

The statistical analysis was performed using Prism 4.0 statistical software (GraphPad software, La Jolla, CA, USA). Chi-square was employed to study the differences in the total number of miRNAs detected in the three groups. ANOVA test was employed to study the differences in mean ΔCT of 2 dysregulated miRNAs (miR-1281 and miR-197-3p) and three stably expressed miRNAs, miR-1234-3p, miR-3656 and miR-3665, in the three cohorts, MPM, WEA and HS. ANOVA test and t-test were employed to confirm the fold-change obtained from ΔΔCt method.

Data for the dysregulated miR 32-3p are presented as a percentage of positive MPM analyzed sera. The 95% Confidence Intervals (CI) of the percentage of positive samples are also reported. Differences among proportions were calculated by Chi-square test for independence in the contingency tables. P value <0.05 was considered to be statistically significant.
